# Validation of the oral health impact profile (OHIP-20sp) for Spanish edentulous patients

**DOI:** 10.4317/medoral.17498

**Published:** 2011-12-06

**Authors:** Javier Montero, Carla Macedo, Antonio López-Valverde, Manuel Bravo

**Affiliations:** 1 Tenured lecturer of Prosthodontics. Department of Surgery. Faculty of Medicine. University of Salamanca. Spain; 2 Postgraduate Student. Department of Surgery. Faculty of Medicine. University of Salamanca. Spain; 3 Associate professor of Periodontology. Department of Surgery. Faculty of Medicine. University of Salamanca. Spain; 4 Professor of Community and Preventive Dentistry. Department of Preventive Dentistry. School of Dentistry. University of Granada. Spain

## Abstract

Objectives: The purposes of this study are to validate the indicator of Oral Health Impact Profile for edentulous patients (OHIP-20sp) in the Spanish population and to analyze the factorial construct of the prosthetic well-being. Study Design: A total of twenty-one (n=21) edentulous patients wearing mandibular implant-over dentures on Locator® (LO) and twenty (n=20) with complete dentures (CD) were retrospectively evaluated in this study. All participants were recruited consecutively and were treated in the previous academic year 2009-2010 by professors of the University of Salamanca. Reliability analyses and validity tests were performed in order to evaluate the psychometric properties of OHIP-20sp employing two different total score methods (additional and simple count). A retrospective evaluation of the impact of the prosthetic treatment was captured with an evaluative instrument derived from OHIP-20, and named POST-OHIP-13. Results: The reliability coefficient (Cronbach’s alpha = 0.91) has shown a high internal consistency. Item-total correlations coefficients ranged from 0.46 and 0.81. Five factors, named as disability, functional comfort, psychosocial impact, pain-discomfort and functional limitations were identified as principal components of the construct, explaining almost 85% of the variance. The 48% of the sample felt at least one impact in an occasional or more frequently manner (generally food packing). The global transition judgment of the prosthetic treatment using the POST-OHIP-13 was significantly higher in group LO than in the CD group. Conclusions: OHIP-20 seems to be a reliable and valid indicator to measure oral impact and satisfaction in the Spanish edentulous population. The underlying construct is comprised by 5 factors named as disability, functional comfort, psychosocial impact, pain-discomfort and functional limitations.

** Key words:**Oral health-related quality of life, edentulous, satisfaction, validation.

## Introduction

In the last National Survey in Spain, it was shown that more than 16% of elderly between 65 and 74 years old had no teeth (fully edentulous) (1). The prevalence of total edentulous in the elderly population varies strongly among populations in Europe (2), being much higher in countries such Iceland (69%), Netherlands (65%) or the United Kingdom (46%). The clinical indices used to describe the oral health status of populations according to standard methodologies of the World Health Organization (WHO) do not say anything neither about the individual’s capacity of performing daily tasks, nor about their physical, psychological and emotional well-being, as well as their quality of life (3) which is where it is remarkably different from the population health of different countries which are better prepared to prevent, diagnose and treat the oral pathology in their populations. Thus, populations could disagree on the prevalence of edentulism, although it is not clear whether they would disagree in terms of well-being associated to it, as people may be rehabilitated with complete denture, or with implant-retained over dentures and others do not restore their teeth at all.

Nowadays it is scientific imperative for the sanataria's to know the benefits for health and well-being that different treatments provided bring to the patient. In dentistry, the development of tools designed to measure the quality of life is relatively recent (4-7) with few studies in which oral quality of life was used as a measure to evaluate how successful or unsuccessful the dental treatment is (8,9). In Spain, two general questionnaires on oral quality of life were recently validated and compared (10): the Oral Health Impact Profile-14 items (OHIP-14) and the Oral Impacts on Daily Performances (OIDP). Nevertheless, these general quality of life indicators are reported by some authors as not being able to address peculiarities of oral impact presented by total edentulous individuals and thus they proposed the use of a specific indicator for edentulous individuals termed OHIP-20 (11) which showed excellent psychometric properties, but still has not been validated for the Spanish population.

The purposes of this study were to validate the OHIP-20 indicator in the Spanish population and to analyze the factorial construct of the prosthetic well-being.

## Material and Methods

Individuals were selected in the School of Dentistry of University of Salamanca in a consecutive sampling from totally edentulous in both jaws treated with conventional dentures or with implant-retained mandibular over dentures in the last academic year 2009-2010. Therefore, 2 prosthetic cohorts were created: Group 1 (n=21) of total edentulous treated with mandibular over dentures on Locator® and a maxillary complete denture (LO: Locator Over dentures) and Group 2 (CD: Complete dentures) of total edentulous treated with conventional dentures (n=20) on both jaws.

All participants received detailed information on the nature of the study which was assessed and approved by the Bioethical Committee of “Universidad de Salamanca” for the clinical investigation, and provided a specific informed consent. Sociodemographic data were collected (age, sex, social class, marital status and residence) as well as some clinical parameters. Questionnaires about prosthetic quality of life (OHIP-20) and the impact of the prosthetic treatment received (POST-OHIP-13) were performed in a face to face interview during one single appointment.

At first, the individuals were asked retrospectively about the main reason for seeking the prosthetic treatment (pain or functional limitation) in the last academic year 2009-2010. A normative prosthesis investigation was also carried out evaluating as satisfying or unsatisfying the following prosthetic factors of both maxillary and mandibular prosthesis: stability, retention, integrity, occlusion and vertical dimension. For this normative evaluation inspection and bilateral palpation was used. Then, patients were asked about the impact on quality of life using the OHIP-20 and the POST-OHIP-13 as an evaluative patient-center measure of the prosthetic outcomes. Satisfaction with mandibular denture and antagonist was also gathered as dichotomies variable.

Linguistic and cultural adaptation

The original version in English OHIP-20 (obtained by the generous mailing of Miss Dr Feine) was adapted to our reference population through two translations carried out independently by two dentists with an advanced level of English, who discussed and produced a consensual Spanish version. The research team made all changes required to improve the intelligibility of OHIP-20 for the target population (Spanish Elderly) and approved the face validity of the consensual version. The conceptual equivalence between the original tool and the consensual version was checked by back-translation made by an independent translator belonging to the Languages Services of the University of Salamanca. The consensual version was piloted in this study.

OHIP-20 instrument

The theoretical basis is grounded on the classification of impairments, comprising cities and disabilities proposed by the World Health Organization and adapted by Locker for dentistry (3). This 20-item instrument, like all those derived from the OHIP-49 (5), focuses on seven dimensions of impact (functional limitation, pain, psychological discomfort, physical disability, psychological disability, social disability and handicap) with participants being asked to respond according to frequency of impact on a 5-point Likert scale coded never (score 0), hardly ever (score 1), occasionally (score 2), fairly often (score 3) and very often (score 4) using a three-months recall period.

Two total impact scoring methods were used. First, the simple scoring method (OHIP-SC) which allows us to calculate the prevalence of impacts on the population for a certain threshold (in our case, frequency = 2). This method calculates the number of items reported as “occasionally” or more frequently. Second, the additive method (OHIP-ADD) is the sum of the scores obtained in 20 items, that is, the higher the score, the higher the frequency of impact is. The OHIP-20 is expected to have at least descriptive capacity of the oral health-related quality of life (OHQoL) for edentulous patients, thus participants can reflect the current state of well being in a transversal manner.

POST-OHIP-13 instrument

For assessing the change in OHQoL after the prosthetic rehabilitation in such retrospective study, the OHIP-20 was conformed in an evaluative design, selecting the main impact-related domains of the prosthetic rehabilitation, based on previous observations of the research team. The POST-OHIP-13 is a 13 items-instrument designed to capture the retrospective global transition judgment of patients about the impact of the dentures on their well being, because the items replies were coded as “better”, “the same” or “worse” due to the prosthetic treatment. The POST-OHIP-13 derived from the original OHIP-20 and the items selected are consecutively: item 1: chewing, item 2: food packing, item 3: satisfaction with diet, item 4: pain-discomfort, item 5: presence of ulcers, item 6: dentures fit, item 7: denture retention, item 8: denture comfort, item 9: smile, item 10: teeth shape, item 11: color and position, item 12: oral well being and item 13: satisfaction with life.

With this trivial approach, it is possible to know if the OHQoL has improved, is the same or has worsened after the prosthetic treatment. In order to quantify intuitively the global effect, items were coded as 1 when they were “better”, 0 for items that were “the same” and -1 for items that were “worse”, so the mean score of these items could give us an idea of the positive or negative effect of treatment. This style of retrospective evaluation has been published elsewhere (12).

Data analysis

In order to study the psychometric characteristics of the OHIP-20 questionnaire, which is a multidimensional tool, we assessed reliability and validity. Reliability should be evaluated by analyzing the scale’s internal consistency, which means that each item assessed different aspects of the same attribute. Reliability was determined by using Cronbach’s alpha value, alpha when eliminating an item and the inter-item and item-total correlation's.

The apparent validity which reflects the understanding of items and content validity were verified for both instruments by asking questions about the difficulties in understanding the items and about situations of impact that had not been mentioned in the questionnaire. Criteria and construct validity were contrasted with the perceived satisfaction towards their mouth and also towards their antagonist prostheses (by using the Student’s T test between fully satisfied versus not fully satisfied). Proportions from both groups of patients with impact were compared using chi-square test.

Bartlett’s sphericity test and Kaiser-Meyer-Olkin (KMO) which are measures of sampling adequacy were used for the factorial structure of the OHIP-20 in order to make the underlying factor structure evident. In addition, a principal component analysis was performed together with the rotation method: Varimax with Kaiser normalization was employed in order to determine underlying dimensions of the prosthetic construct. Items were attributed to rotated factors when they had only one load factor of 0.5 or more. All analyses were performed with the use of SPSS (v15) (Statistical Package of Social Sciences.SPSS. Inc. Chicago, IL).

## Results

The sample is comprised by 41 fully edentulous patients, with a mean age of 68.0 ± 11.7 months, mainly women (58.1%), pensioners (71%) who lived in the urban area of Salamanca. The main reason for seeking prosthetic treatment was functional limitation (65.9%), and pain (34.1%). Out of 41 individuals, 21 were in Group 1 (LO: Locator Over dentures) and 20 were in Group 2 (named CD: complete dentures). Both groups (LO and CD) were found to be comparable in sociodemographic terms and in the main reason for seeking prosthetic treatment ( Table 1). The normative assessment revealed that there were significant differences among groups but only for mandibular prosthesis, being the proportion of satisfactory cases much higher within the Locator Group, and mainly in retention and stability ( Table 1).

**Table 1:**
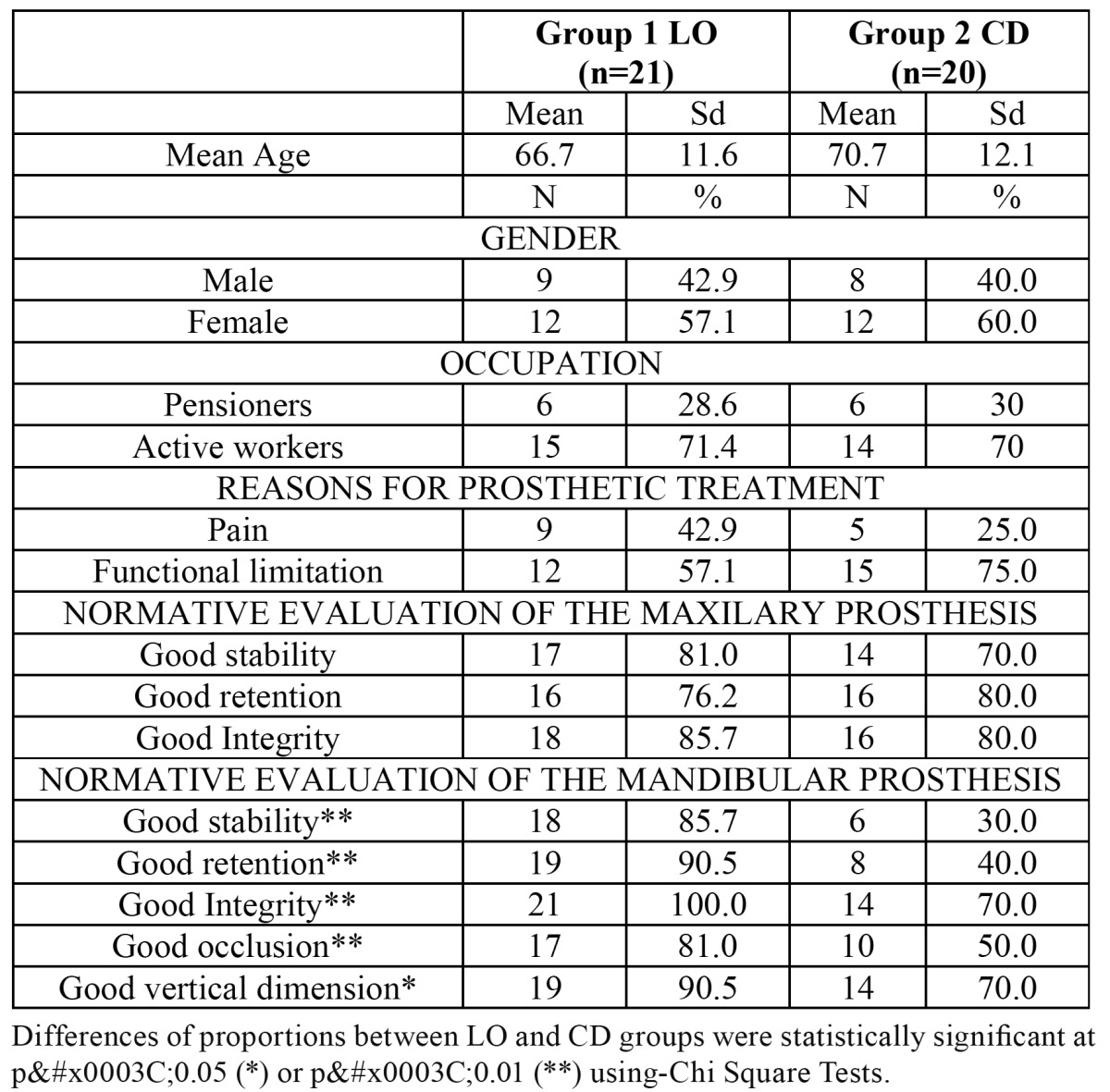
Sociodemographic characteristics, reasons for seeking prosthetic treatment and normative evaluation of the prosthesis among the subgroups (LO and CD) of the sample (n=41).

The analysis of inter-item correlation's showed a distribution of positive inter-item correlation's, except for the negative correlation (0.09) between “displeasure” and “easily irritated by others” ( Table 2). The correlation of each item with a total score ranged from 0.46 to 0.81 ( Table 3). This analysis showed the value for Cronbach´s alpha with standardized items of 0.91. The apparent and content validity were checked since all individuals from the study declared and that they understood all items and that they did not mention any situation of impact that was not collected by the pilot OHIP-20. The criterion and construct validity were proven by detecting that those individuals who were satisfied with their mouth (n=33, 80.7%) had a lower total additive score (9.6 ± 12.6) than counterparts (17.3 ± 8.6).

**Table 2:**
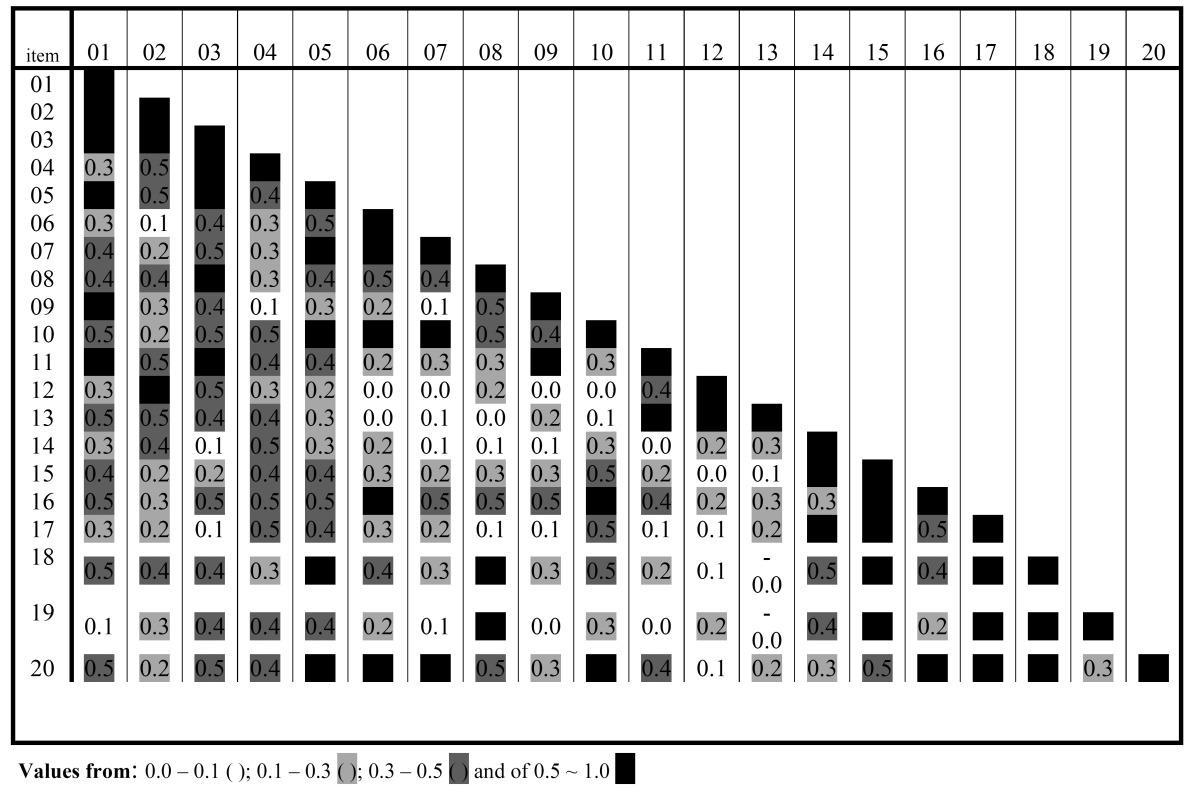
Analysis of reliability of OHIP-20: Distribution of inter-item correlations (n=41).

**Table 3:**
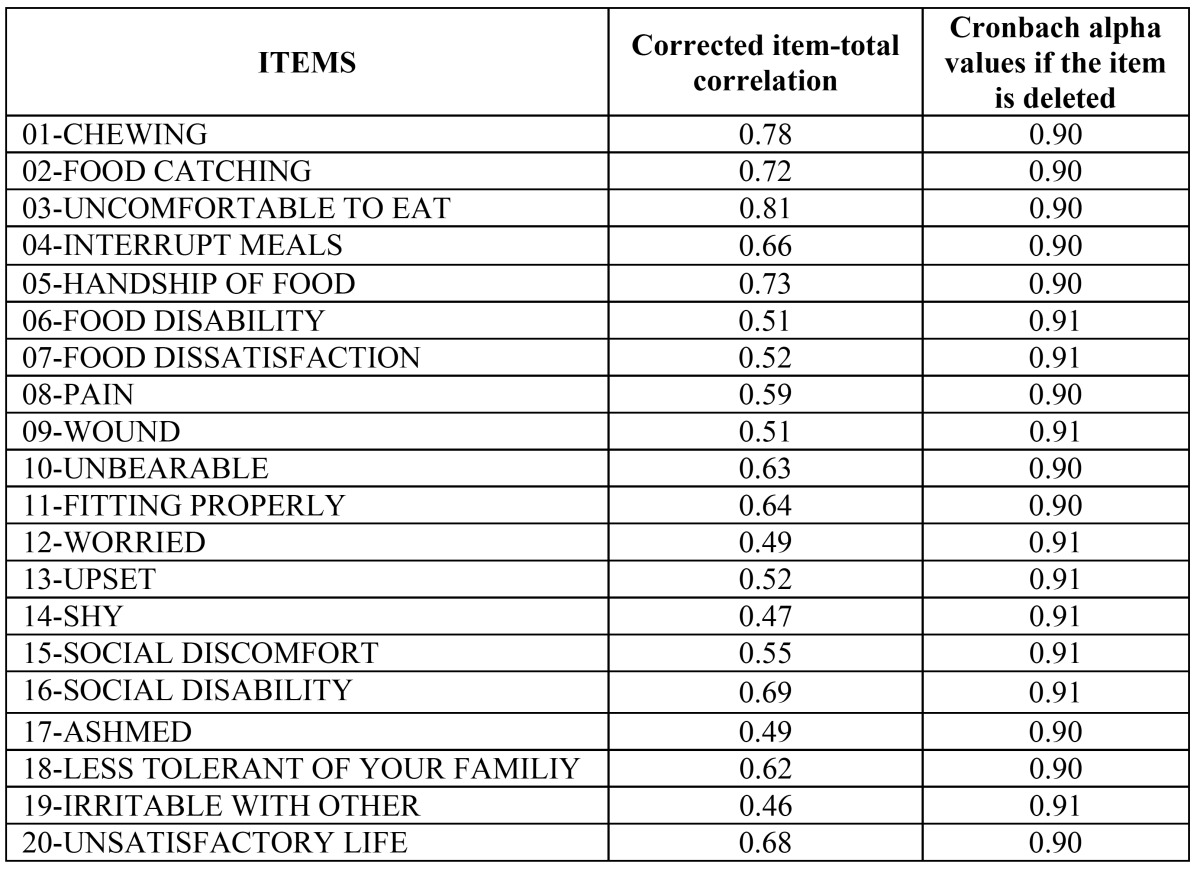
Analysis of reliability of OHIP-20: item-total correlations and alpha value if the item is removed (n=41).

In order to show the factor’s underlying structure, Bartlett’s Sphericity test was performed. Results 

(χ2 =867.479, p<0.0001) suggested that there were latent factors. KMO measure has produced a global value of 0.38.

Factorial analysis has revealed five dimensions with eigenvalues above 1, that explain 84.7% of variance and according to the items loading could be named as disability, functional comfort, psycho social impact, pain-discomfort and functional limitations ( Table 4). On the rotated component matrix, in which each item’s load is seen over 5 factors, that is, for each item’s influence on each factor a threshold of ≥0.5 was used as a substantial load indicator.

**Table 4:**
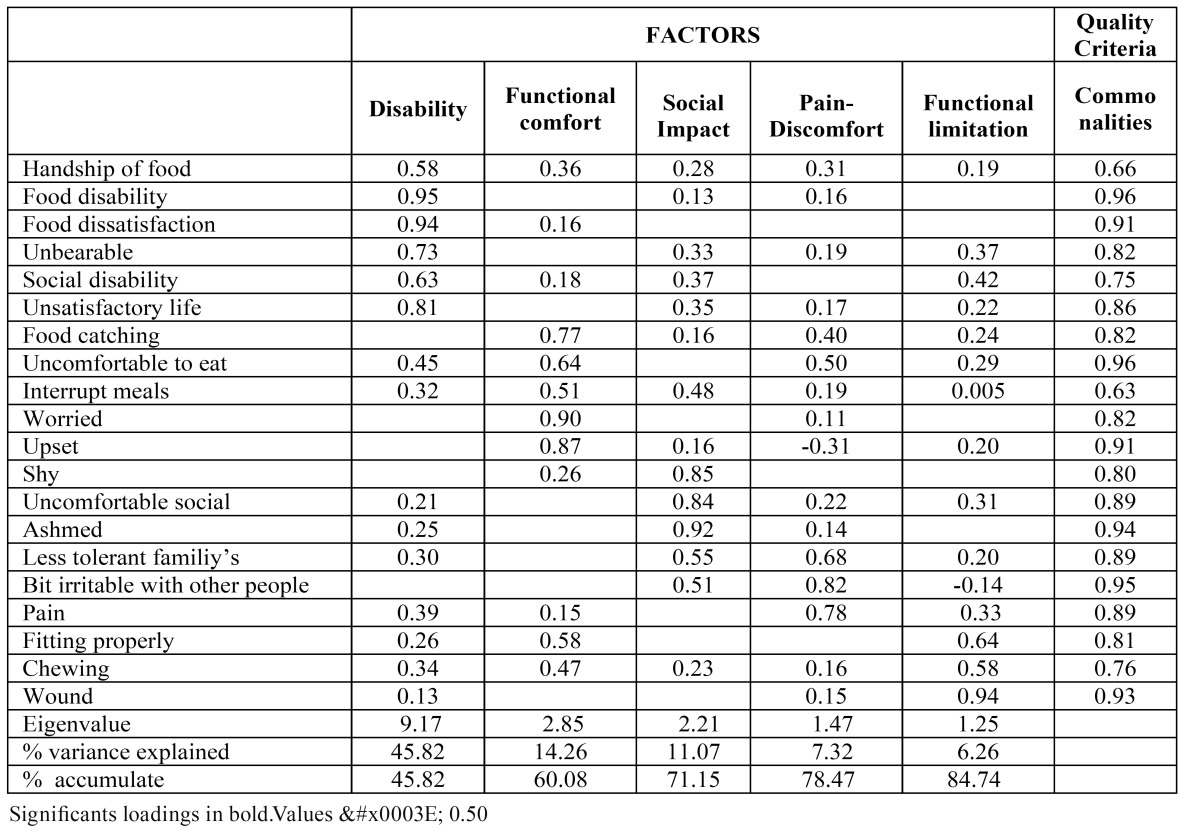
Factorial structure: Factor loading on the rotated matrix of the OHIP-20 (n=41).

**Figure 1:**
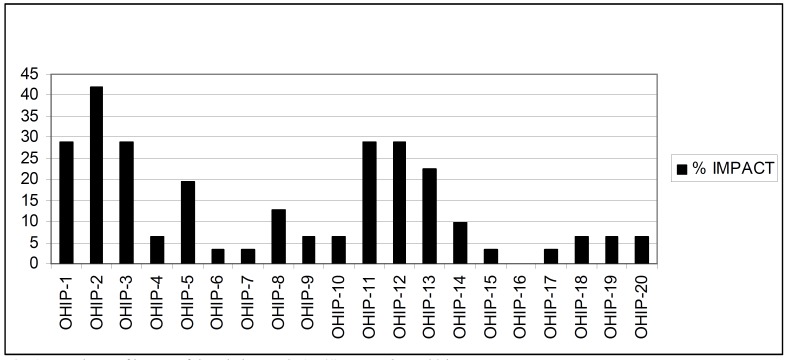
Prevalence of impact of the whole sample (n=41) among OHIP-20 items.

The majority of subjects were satisfied with their conventional dentures (60.0%) and their antagonists (75.0%), and within LO Group all participants were satisfied with their over denture and most of them were also satisfied with their antagonistic (81.0%). However the prevalence of impact was 48.4% (% subjects reporting at least one item affected as occasional or more frequently manner), and the OHIP-ADD total additive score was 11.1 ± 12.2. The OHIP-SC score was 2.7 ± 3.6 (average of items affected in an occasional or more frequently manner). The most affected item was OHIP-2 (food packing) affecting to 41.5% of the sample (Fig. 1).

**Figure 2:**
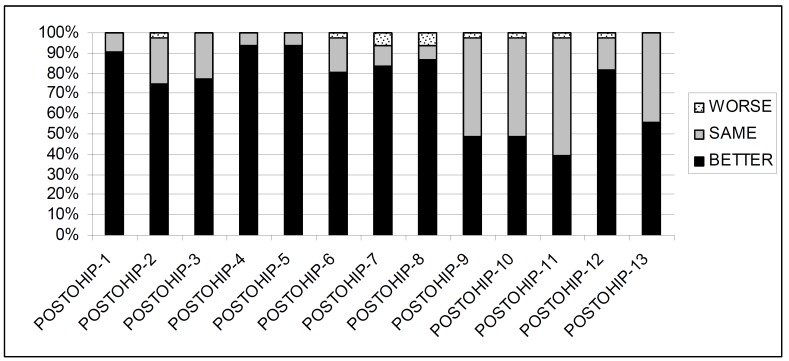
Distribution of the evaluative Post-OHIP scores among the sample (n=41).

Prosthetic treatment improved on average 9.3 ± 3.8 items, which is the Additive Total Score of the 13 items the POST-OHIP-13 (Fig. 2). Item 7 (denture retention) and item 8 (denture comfort) were worsened in 6.5% of the sample. There were not statistically significant difference among subgroups in the impact of OHQoL using the OHIP-20-ADD (LO: 9.8 ± 13.2; CD: 13.7 ± 10.) or the OHIP-20-SC (LO: 2.6 ± 4.1; CD: 3.1 ± 2.6), but the global transition judgment using the POST-OHIP-13 was significantly higher in group LO (10.4 ± 2.1.) than in the CD group (6.8 ± 4.4).

## Discussion

Procedures of linguistic cultural adaptations are a critical component of the validation process of a tool which was aimed at another target population. In the present study, the process of translation from English into Spanish was direct and the comparison between the original OHIP-20 and the backward-translated English version has not shown conceptual differences. Equivalent words which were needed to translate the questionnaire were not difficult to be found due to the simple structure used in the OHIP-20 and to the universal nature of its content. This study is, as far as we know, the first to use OHIP-20 in the Spanish population and the first assessing subjectively and normatively the prosthetic rehabilitation of the edentulous patients in Spain.

The sample size (n=41) was similar to other studies with the same methods and objectives (11,13-15), but it seems insufficient to detect significant differences between groups in relevant issues, like OHIP-ADD and OHIP-SC, as happened in other studies (14). It seems that the minimal sample size per subgroup for discriminate using both scoring methods of the OHIP-20 should be n=50, as found other authors (13). However as our main objective was to validate the instrument among edentulous patients and to evaluate the underlying construct, authors believe that our results support this purpose. Another limitation of the study is the lack of test-retest or interclass correlation analysis to corroborate the stability of the score given by subjects or examiners, respectively. This issue should be necessarily tested in future research

As expected, the normative evaluation of the maxillary prosthesis was mostly satisfactory for both groups, but significant differences were found only for the mandibular prosthesis (LO vs CD), and this fact could be the reason behind the lower improve ent perceived after treatment for the CD group against the LO group found in this study and reported elsewhere (13). The normative evaluation of the prosthesis carried out in this study is a trivial way to justify clinically why satisfaction and OHQoL should vary among subgroups or individuals, as reported elsewhere (16). This approach should encourage researchers to combine subjective and normative assessment of the treatment, because it could offer useful insights about the clinical significance of our work.

Within the limitations of this study, it could be stated that the Spanish version of the OHIP-20sp seems to be valid and reliable for use in the total edentulous population in Spain. Internal reliability results (inter-item and item-total correlations) in which all item-total correlations were over 0.2 supported the consistency of the items in the scale ( Table 2). The standardized Cronbach’s alpha value for the sample was 0.91, which is considered excellent and higher than reported elsewhere (14,15), and in addition, this value does not increase when eliminating an item thus all items should remained in the scale ( Table 3).

Construct and criterion validity were mainly supported by using subjective criteria such as satisfaction with the mouth, as recommended several authors’ recommendations (17-19), because the indicators of quality of life are designed to measure health according to a holistic concept in which more and more psychological and sociological characteristics are recognized and which may be only expressed by subjective feelings.

The use of the POST-OHIP-13 instrument eases the interpretation of the change scores. Our treatment should be perceived by patients in a simple way: better, same or worse. There are other methods to estimate such changes, as the minimal important difference in prosthodontics (20), but the proposed method is easier and cheaper, and compulsorily indicated for retrospective evaluations (12). The self assessment of the prosthetic outcomes through the POST-OHIP-13 (Fig. 2) shows that a positive prosthetic effect was mainly registered on items 1,4,5,6,7,8 and 12 corresponding to “chewing”, “pain-discomfort”, “presence of ulcers”, ”dentures fit”, “denture retention”, “prosthetic comfort” and “well-being with their mouths” respectively, which are the main complaints reported by edentulous patients seeking treatment (16). However the prosthetic treatment was not able to resolve the food packing behind prosthesis (Fig. 1) because it was a common condition among edentulous patients (12).

Even if experts’ opinions may be valid to preliminarily add items to conceptual factors, the statistical explanation based on factorial analysis allows us to view the construct and the interaction among variables (21). The factorial structure found in this study met some quality criteria. First, KMO’s global value of 0.38 suggests the sample was suitable to search for underlying factors and that all factors may play a role in this search with a considerable statistical weight. The commonality analysis showed that all items contribute to the variability of the proposed factorial solution. All of them exceed a threshold of 0.5 which shows that the items are valid for the factorial solution suggested. Furthermore, the factorial structure achieved corroborates the underlying theory in which the construct is conceived (22). There are five factors which explain 84.7% of the variation, each factor was evaluated for more than two items, reducing the malign effect of a certain individual value, and the loads of each item of the factors had eigenvalues above 1 (23,24). There is a general consensus in the scientific literature regarding the multidimensional nature of the oral quality of life, but there is no consensus regarding factorial solutions, even when physical and psychological factors and social well-being were highlighted (25). Some general questionnaires dealing with OHQoL identified a set of dimensions from the factorial solution which might easily be matched with the ones offered in the present research (21,26).

In recent years, the presence of physical, psychological and social factors in the construct of the edentulous population well-being has been confirmed (27). Our factorial solution corroborates that prosthetic quality of life is multidimensional, but the structure is not parsimonious (in fact, there are impact-related items loading on distinct factors) and author are aware that factors have been named arbitrarily.

Further efforts should be made by clinicians in order to investigate the effect of distinct treatments modalities on patients’ satisfaction and OHQoL.
